# Publisher Correction: Tryptophan C-mannosylation is critical for *Plasmodium falciparum* transmission

**DOI:** 10.1038/s41467-022-32664-8

**Published:** 2022-08-23

**Authors:** Sash Lopaticki, Robyn McConville, Alan John, Niall Geoghegan, Shihab Deen Mohamed, Lisa Verzier, Ryan W. J. Steel, Cindy Evelyn, Matthew T. O’Neill, Niccolay Madiedo Soler, Nichollas E. Scott, Kelly L. Rogers, Ethan D. Goddard-Borger, Justin A. Boddey

**Affiliations:** 1 The Walter and Eliza Hall Institute of Medical Research, 1 G Royal Parade, Parkville, VIC 3052 Australia; 2 Department of Medical Biology, University of Melbourne, Parkville, VIC 3010 Australia; 3 Department of Microbiology and Immunology, University of Melbourne at the Peter Doherty Institute for Infection and Immunity, Parkville, VIC 3010 Australia

**Keywords:** Parasite biology, Glycobiology

Correction to: *Nature Communications* 10.1038/s41467-022-32076-8, published online 29 July 2022

The original version of this Article incorrectly cited reference 85 instead of reference 84 in the methods section ‘An. stephensi immune-responsive gene expression’. This has now been corrected in both the PDF and HTML versions of the Article.

