# Supplementary material for: Tryptophan C-mannosylation is critical for Plasmodium falciparum transmission
Source: Nat Commun. 2022 Jul 29;13:4400. doi: 10.1038/s41467-022-32076-8 (PMC9338275; doi:10.1038/s41467-022-32076-8)
Supplement: Supplementary file 1 — Supplementary Information [file 41467_2022_32076_MOESM1_ESM.pdf]

Supplementary information

Tryptophan C-mannosylation is critical for *Plasmodium falciparum* transmission

```
PfDPY19   ----------------------MKKNKISLFIISSLVCAFVTL-TFYKRY---RYGFDRN   34
CeDPY19   MAKKPKNSPEKSKYSSDTSSSLYSQTWLASVVIIGLLVGYINYQHVYTLFENDKHFSHLA   60
                                 .:. :: .:* .*: .::.   .*. :   ::  .

PfDPY19   YVEQKLSLYSEESFYFSFYNDIVKSNTFGEGINYLLKDNRSEYPDTINAIKRFNIYPEII   94
CeDPY19   DFEREMAYRTEMGLYYSYYKTIINAPSFLEGVQEITHDTVTEHGHEINTLNRFNLYPEVI   120
           .*::::  :* .:*:*:*: *::: :* **:: : :*. :*: . **:::***:***:*

PfDPY19   LGALWKGLNLE-------------------------SYILTPYNFYVYAVIFLQAASVSV   129
CeDPY19   LAFLYRPFRAFAKSANWQIELCWQVNRGELRPVESCEGIGNPHYFYITGVFIVAGTVASS   180
          *. *:: :.                           . * .*: **: .*::: .: .*

PfDPY19   LFFFSVYIGKSYFPGVIFLMLFFSCFREKFIMRLSAFPLRENFASVFMWCNIILIYIILK   189
CeDPY19   IFYLGVLVSDSIFGGFLSVLCFAFNHGE-ATRVQWTPPLRESFAFPFIIGHIAILTFVIK   239
          :*::.* :..* * *.: :: *   . *       : ****.**  *:  :* :: :::*

PfDPY19   DKEIPILKYIGLFFSSLLCFLFWQFSVFVSVTHIVSLFIV---DLLGYNIINKLNN---I   243
CeDPY19   YKKSGHSMILLLTSMAVPALLFWQFTQFAFFTQICSIFLAFSLDLIPFSTAKTVIHSHII   299
           *:      : *   :: .:*****: *. .*:* *:*:.   **: :.  :.: :   *

PfDPY19   LFI----FCFSYLLSITLTFFPRYLLCTYFPYVLIAILITNVIFNYFSKKKKDDVNNNNN   299
CeDPY19   SFLIGFLLLFGNEMMITALYFPSILALGMIIYI--SPLLSNLKFR---------------   342
           *:    : *.  : **  :**  *    : *:  : *::*: *.

PfDPY19   NNNIYNMNETNNQHSKTCTFVRTTKEKNHEMINNNKINNLKNNNNHPCNNYNSYNQCEER   359
CeDPY19   ------------------------------------------------------------   342

PfDPY19   KNEDINDKPTNKYNKEISLFTLFKNLYIIKNWIFILKKGLTSIFIFLILRLIIFSKDKDD   419
CeDPY19   -----------------------------PAYVLFLAIIFASITLGLKIGLSKGLGIEDD   373
                                         ::::*   ::** : * : *      :**

PfDPY19   SHVISLLKVRLKLANHNFDTMLYSSGSEFNPFSKYMFHMLKESAVYEYFIIFNILFFIYI   479
CeDPY19   AHIFDILRSKFT-SFANFHTRLYTCSAEFDFIQYSTIEKLCGTLLIPL-ALISLV--TFV   429
          :*::.:*: ::. :  **.* **:..:**: :.   :. *  : :     ::.::   ::

PfDPY19   LNYCKQ--LL-KGKQTQQYHIFKSSFIFLIYQLVFFILLMLIISRLRVLALPLICLFSSL   536
CeDPY19   FNFVKNTNLLWRNSEE---IGENGEILYNVVQLCCSTVMAFLIMRLKLFMTPHLCIVAAL   486
          :*: *:  ** :..:       :..::: : **    :: ::* **:::  * :*:.::*

PfDPY19   VGSPHFLDDLYFLTSQNFLKSKRTNKGRLHKIIIFSICLVQCAYPFKKYFPQYEYMNMIN   596
CeDPY19   FANSKLLGGD-----------------RISKTIRVSALVGVIAILFYRGIPNIRQQLNVK   529
          ... ::*..                  *: * * .*  :   *  * : :*: .    ::

PfDPY19   NEPINLQKNLDLIIWLKKNIKEGEALISDIPTSSFLRCTTNYKFVLHPQYEDSNLRKRVQ   656
CeDPY19   GE-YSNPDQEMLFDWIQHNTKQDAVFAGTMPVMANVKLTTLRPIVNHPHYEHVGIRERTL   588
          .*  .  .:  *: *:::* *:. .: . :*. : :: **   :* **:**. .:*:*.

PfDPY19   DYYMFSACLPFSDGKKYIFEKYKSRYFISNIYRCSSSGSKINVFTISDKIDSNYARCEKK   716
CeDPY19   KVYSMFSKKPIAEVHK-IMKEMGVNYFVFQLMNCSNDERRPECVYRG-MWDEED---PKN   643
          . * : :  *::: :* *:::   .**: :: .**.. :  : .  .   *.:     *:

PfDPY19   RKTMRFCNRVLYDDKNYKTLFRNGKFSVIYFTPEIIPDNTPYKYFNQKKYSNIIYYEPWI   776
CeDPY19   SGRTALCDLWILAAN-SKDNSRIAPFKIVYNAN------R--NYIVLKI-----------   683
               :*:  :   :  *   * . *.::* :          :*:  *

PfDPY19   KRCMLTDDKCALHITEVARTYLDMLKYNLIAFTLYDYVENNLLHNNVEVIFHIAEYYDYD   836
CeDPY19   ------------------------------------------------------------   683

PfDPY19   KKDHKKANELYRKAINLIIKKESDLGTYIIGQTPYVSIPRKIQILSSFLYFIVDMSLYKD   896
CeDPY19   ------------------------------------------------------------   683

PfDPY19   RQEILLIYKNMNEFINAALFALDNRFYYEIIKSTQPNQKDQIIKRSFYKKELHTVINALC   956
CeDPY19   ------------------------------------------------------------   683

PfDPY19   QNTIYLKQIQHEHFQYIHIYNNLWTLIKRLTHLENCVIENLAIYENRKIRFLDYLLFFYI   1016
CeDPY19   ------------------------------------------------------------   683

PfDPY19   YN 1018
CeDPY19   -- 683

identity   (142/1082) 13.1%
similarity (398/1082) 36.8%
```

Supplementary Figure 1. Sequence alignment of DPY19 from *P. falciparum* (PF3D7_0806200) and *C. elegans* (CCD62139.1), showing identical (dark grey), similar (light grey) and essential residues for enzyme activity (red).

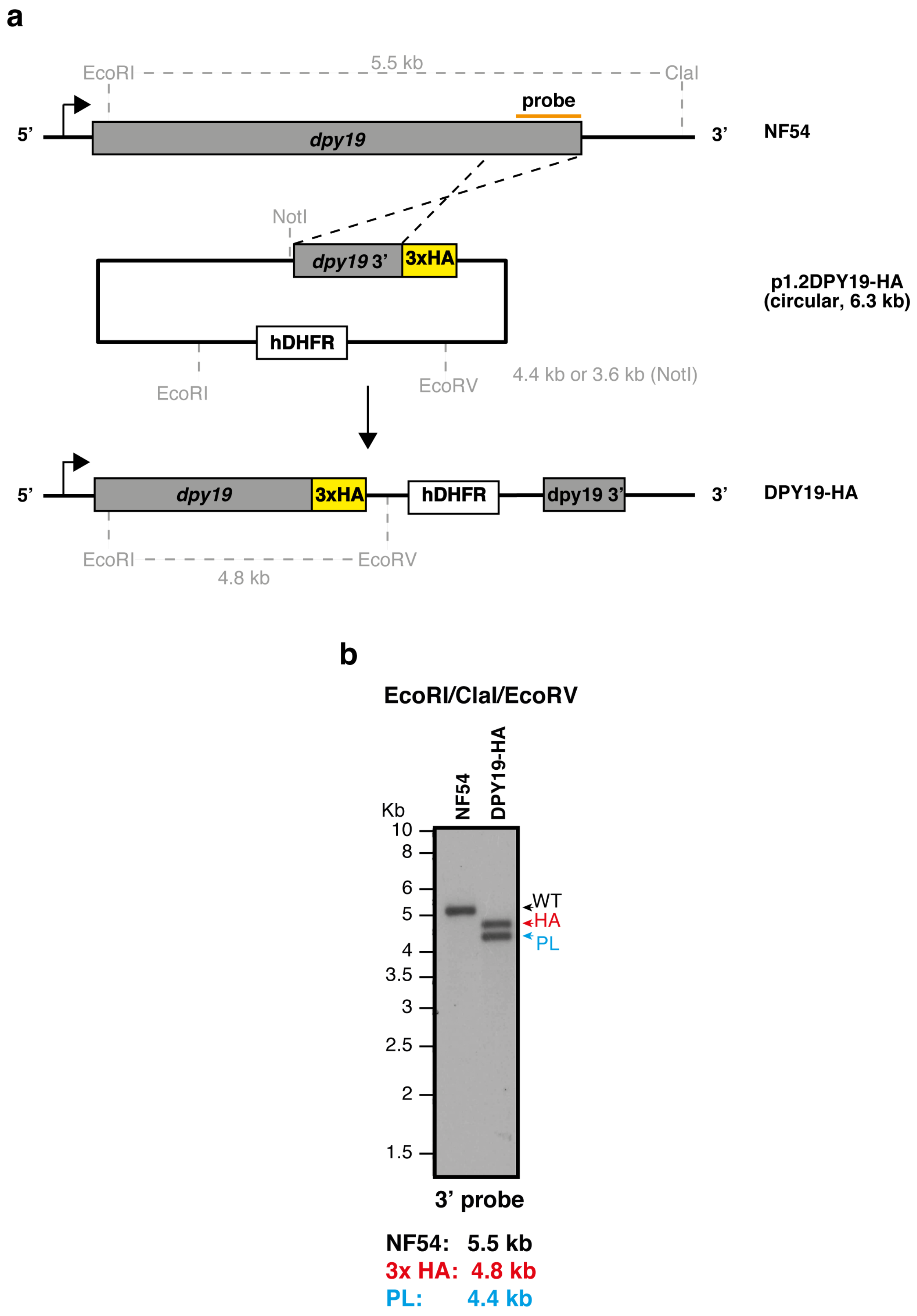

Supplementary Figure 2. a Strategy for inserting a C-terminal triple hemagglutinin (HA) epitope tag into the *P. falciparum DPY19* locus. **b** Southern blot analysis of parental NF54 and DPY19-HA parasites after digestion of genomic DNA with *Eco*RI/*Cla*I/*Eco*RV. The probe used (orange bar) for genotyping and the expected fragment sizes after digestion of the loci is shown. Data represent n=1 experiment confirming integration.

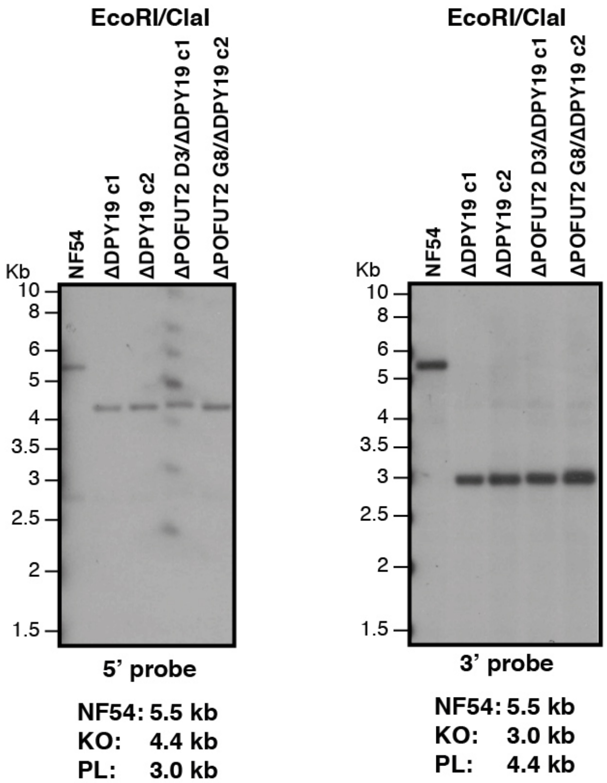

Supplementary Figure 3. Southern blot analysis of parental NF54, Δ*DPY19* and Δ*POFUT2*/Δ*DPY19* clones after digestion of genomic DNA with *Eco*RI/*Cla*I. The NF54 and mutant band sizes are as expected indicating all mutant clones have the Δ*DPY19* locus. Data represent n=1 experiment confirming independent knockout clones.

a

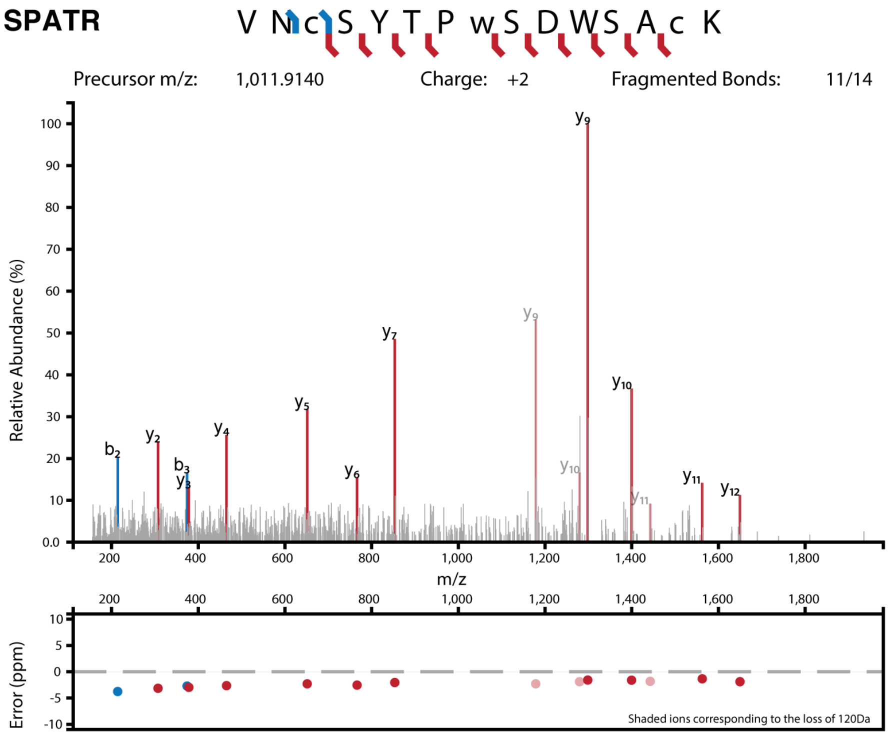

b

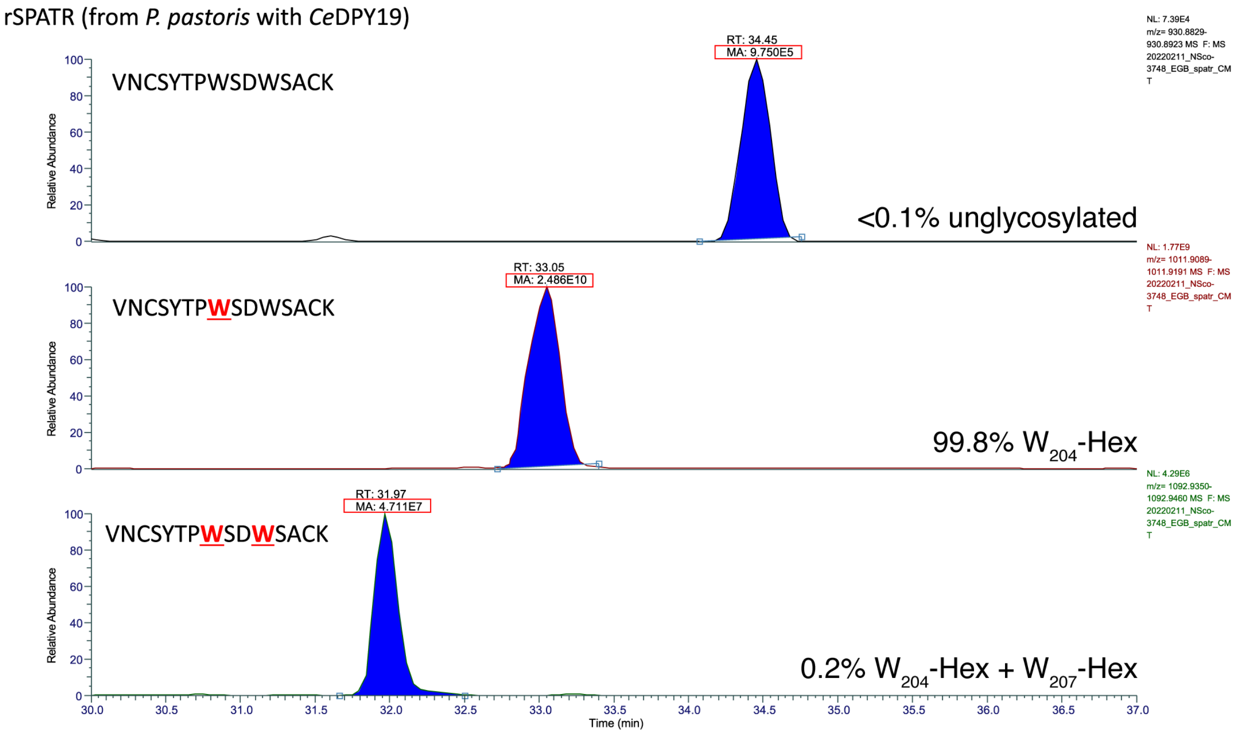

Supplementary Figure 4. Occupancy and localization of tryptophan C-mannosylation on rSPATR. **a** Tandem mass spectra of the most abundant C-mannosylated peptide from rSPATR enabled localization of the modification to the first tryptophan (W204) of the WxxW motif. **b** EIC for the unglycosylated, mono-glycosylated, and di-glycosylated peptides from rSPATR. The relative abundance of each peptide was estimated from the integrals of each peak.

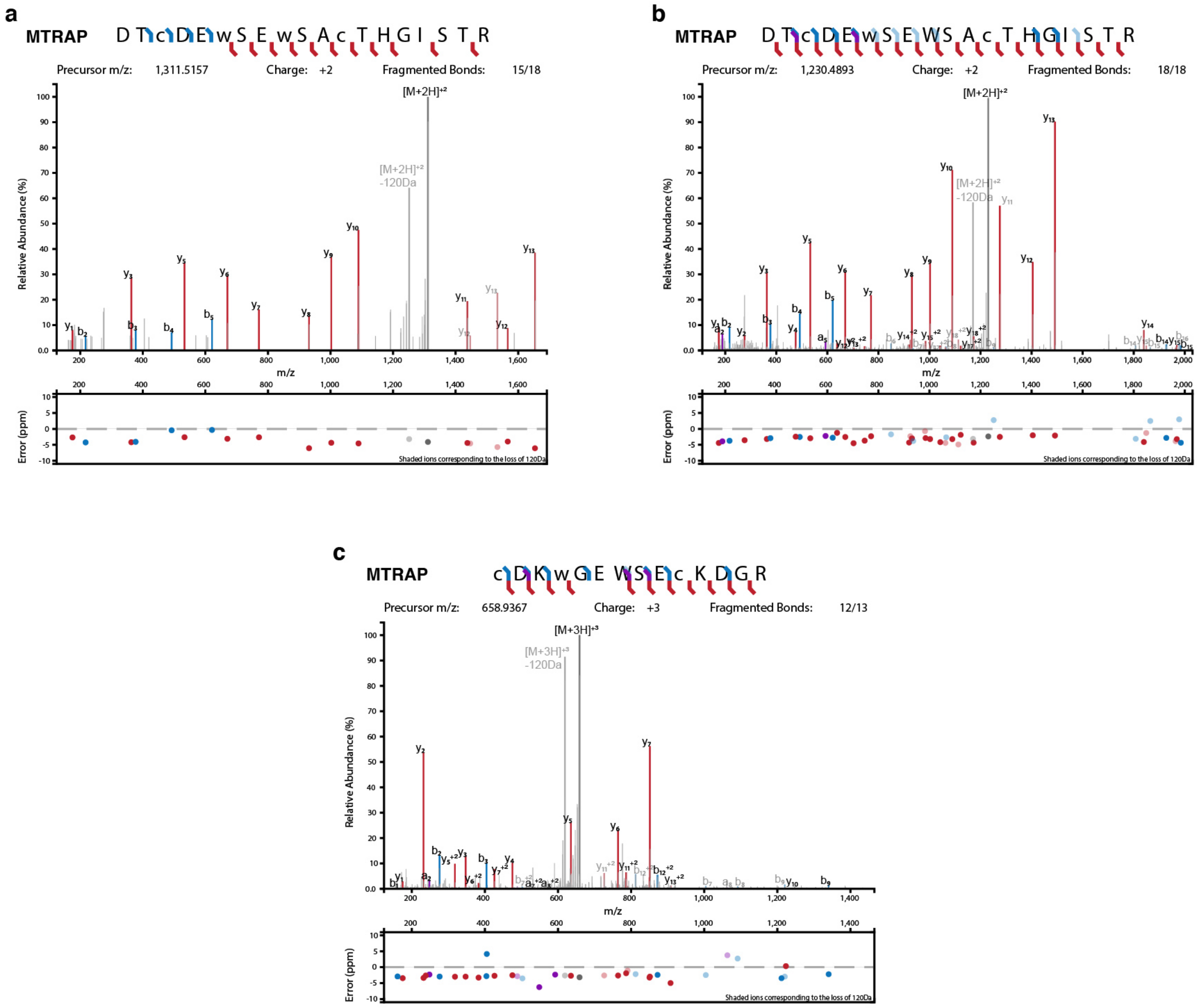

Supplementary Figure 5. Localization of tryptophan C-mannosylation on rMTRAP. Tandem mass spectra of C-mannosylated peptides from rMTRAP for the **(a)** di-glycosylated peptide DTCDEWSEWSACTHGISTR and **(b-c)** mono-glycosylated peptides DTCDEWSEWSACTHGISTR and CDKWGEWSECKDGR.

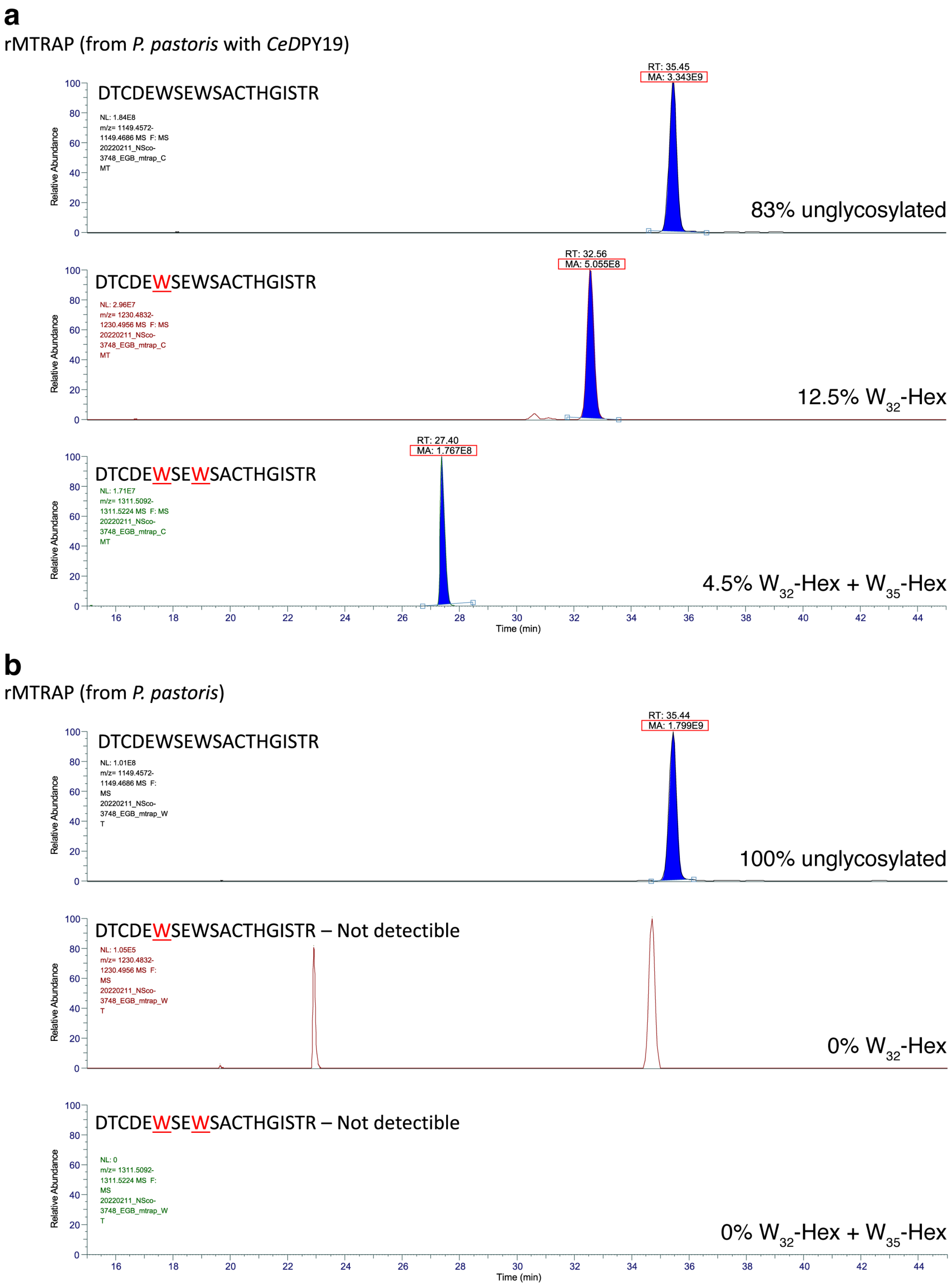
a
rMTRAP (from P. pastoris with CeDPY19)
DTCDEWSEWSACTHGISTR
RT: 35.45
MA: 3.343E9
83% unglycosylated
DTCDEWSEWSACTHGISTR
RT: 32.56
MA: 5.055E8
12.5% W32-Hex
DTCDEWSEWSACTHGISTR
RT: 27.40
MA: 1.767E8
4.5% W32-Hex + W35-Hex
Relative Abundance
Time (min)
b
rMTRAP (from P. pastoris)
DTCDEWSEWSACTHGISTR
RT: 35.44
MA: 1.799E9
100% unglycosylated
DTCDEWSEWSACTHGISTR – Not detectible
0% W32-Hex
DTCDEWSEWSACTHGISTR – Not detectible
0% W32-Hex + W35-Hex
Relative Abundance
Time (min)

c

rMTRAP (from *P. pastoris* with *Ce*DPY19)

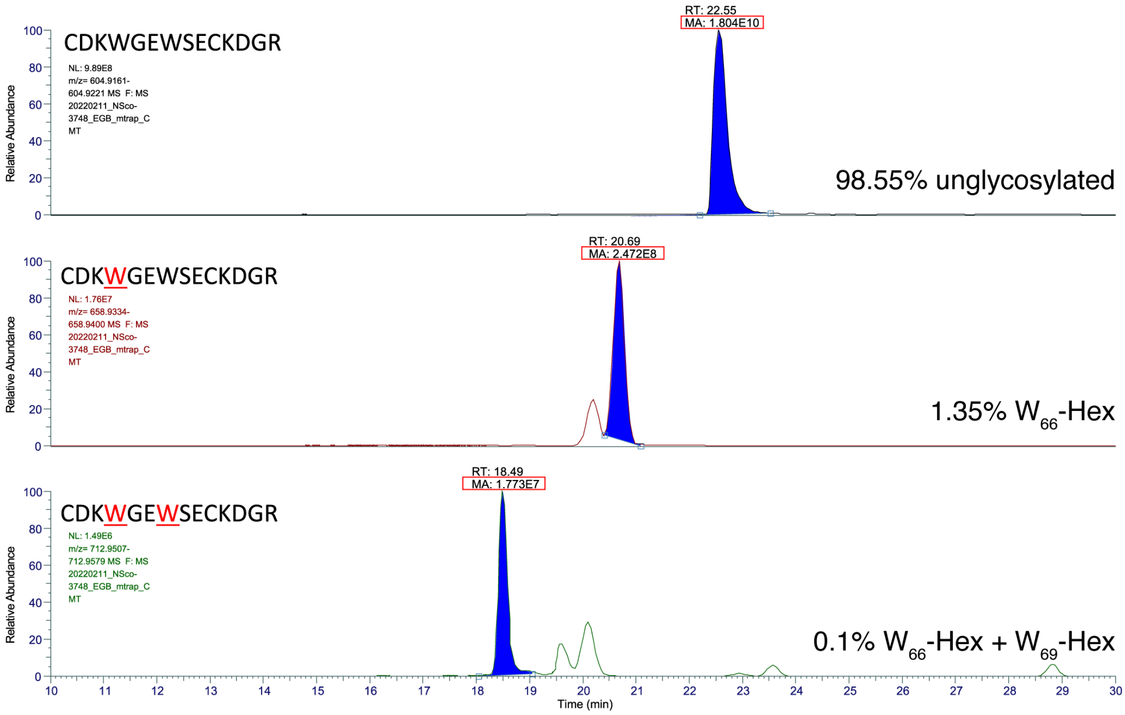

d

rMTRAP (from *P. pastoris*)

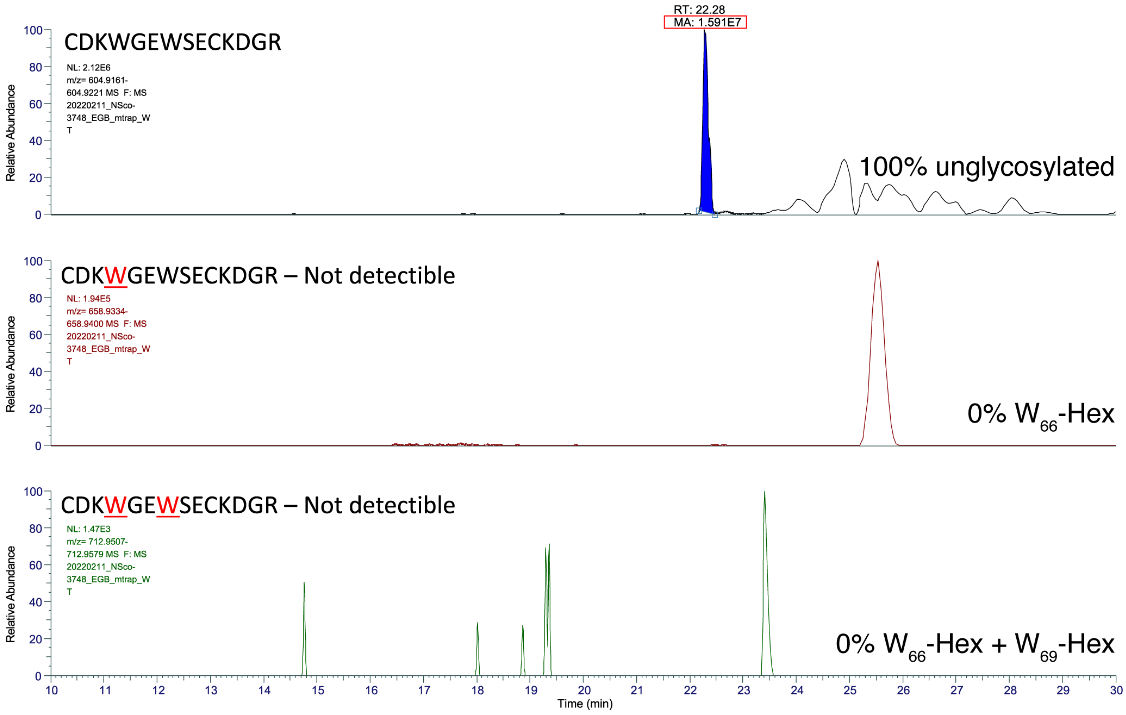

Supplementary Figure 6. Occupancy of tryptophan C-mannosylation on rMTRAP. **a-b** EIC for the unglycosylated, mono-glycosylated, and di-glycosylated DTCDEWSEWSACTHGISTR peptide from rMTRAP. The relative abundance of each peptide was estimated from the integrals of each peak. **c-d** EIC for the unglycosylated, mono-glycosylated, and di-glycosylated CDKWGEWSECKDGR peptide from rMTRAP. The relative abundance of each peptide was estimated from the integrals of each peak.

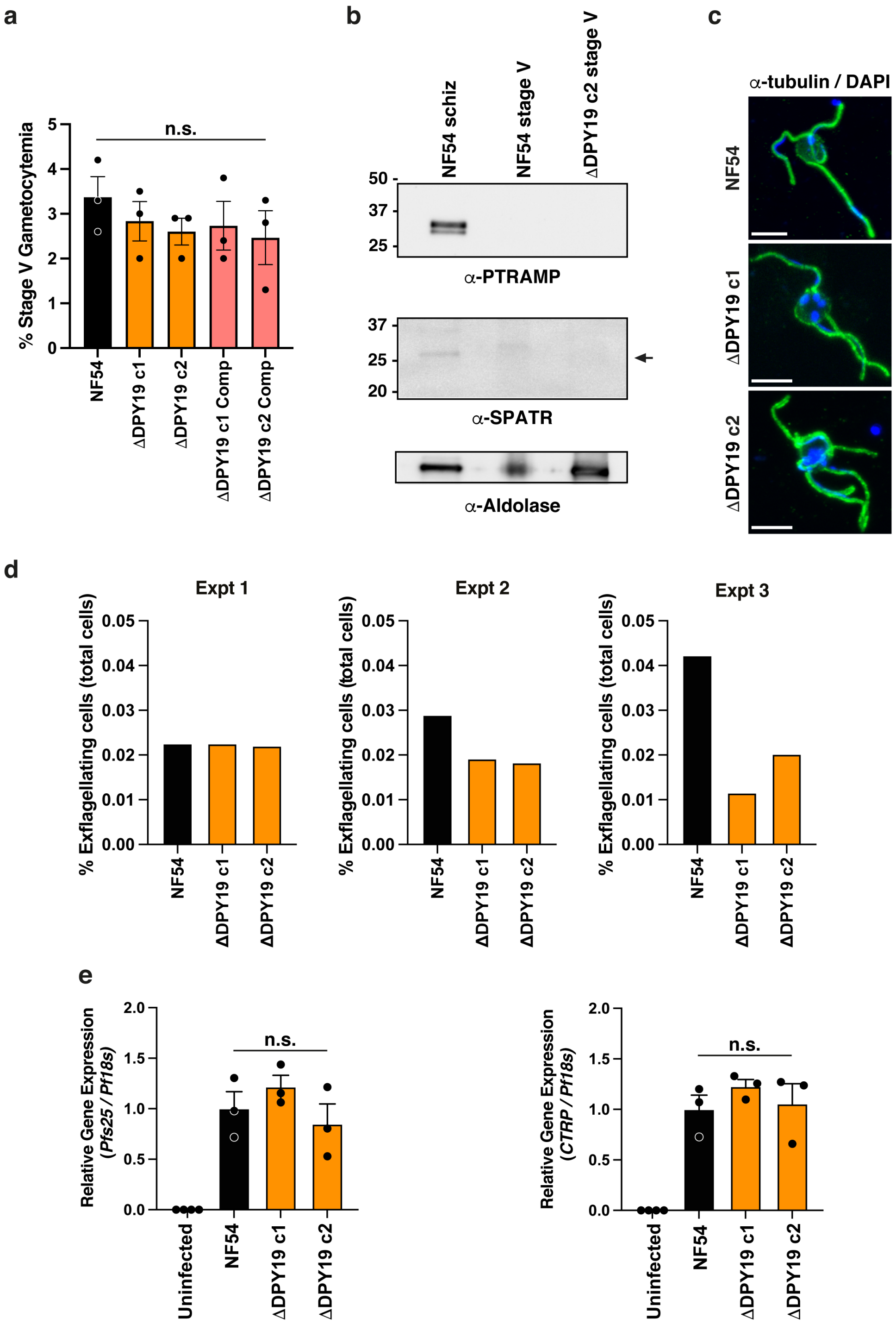

Supplementary Figure 7. a No difference in stage V gametocytemia was observed between NF54, Δ*DPY19* and Δ*DPY19* parasites. Data are mean ± s.e.m. from n=3 experiments and were compared by one-way ANOVA (Kruskal-Wallis test). n.s., not significant. **b** Detection of PTRAMP and SPATR expression by immunoblot in asexual schizonts (schiz) but not in stage V gametocytes. Aldolase loading control is ~40 kDa. Data represent n=2 independent experiments. **c** Exflagellation by NF54 and Δ*DPY19* microgametes. Scale bar, 5 mm. **d** Quantification of exflagellation by NF54 and Δ*DPY19* microgametes using light microscopy, represented as a percentage of total (infected and uninfected) erythrocytes. n= 3 experiments. **e** RT-qPCR quantification of *P. falciparum Pfs25* and *CTRP* mRNA expression relative to housekeeping gene *P. falciparum 18S* in midguts from mosquitoes that were sugar fed (uninfected) or fed infected blood containing NF54 or ΔDPY19 clones. Data are mean ± s.e.m. from n = 25 midguts per condition per experiment from 3 independent experiments, pooled and analyzed by a one-way ANOVA using a Dunnet's test. n.s., not significant.

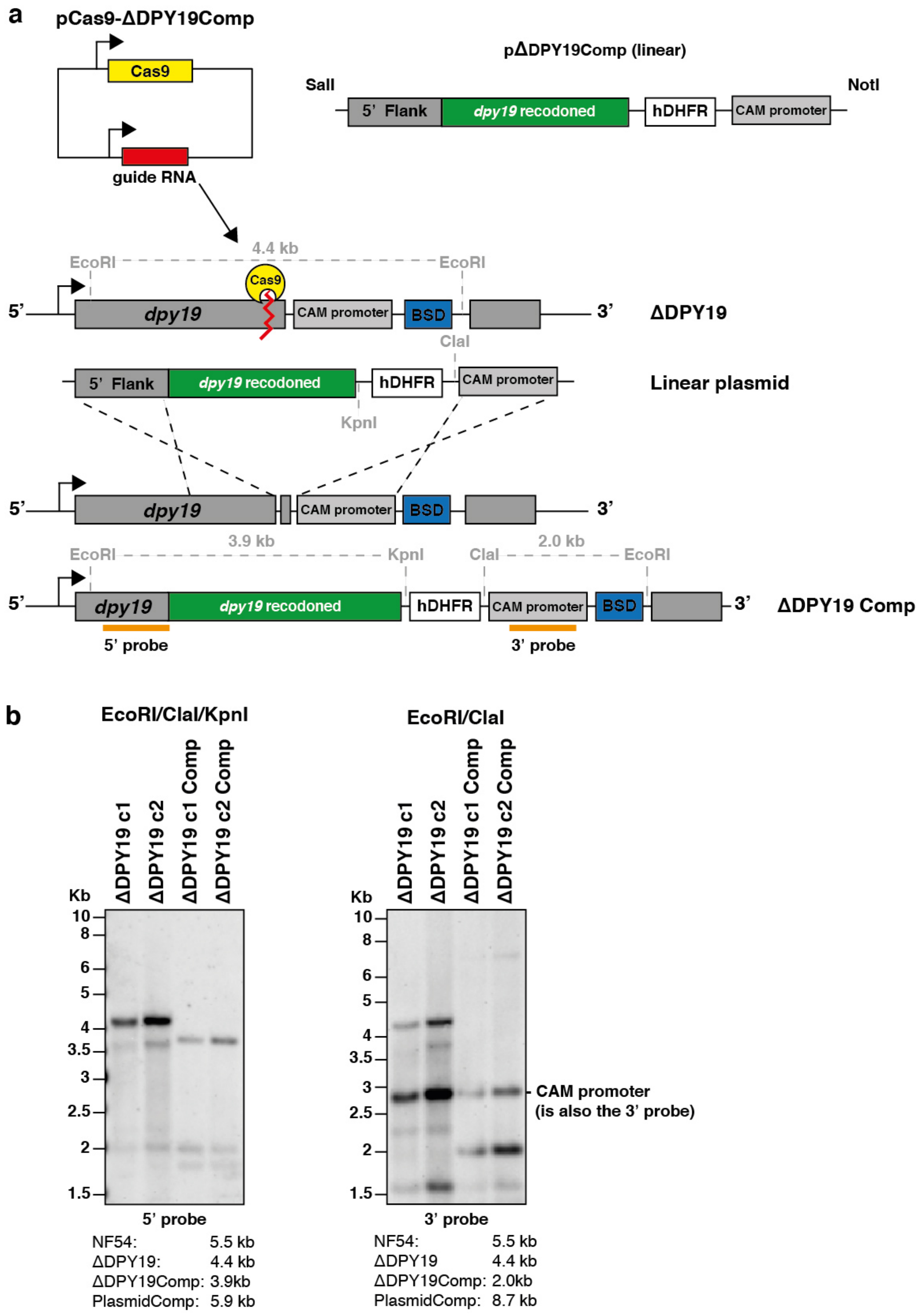

Supplementary Figure 8. Complementation of Δ*DPY19* parasites with the *DPY19* gene to restore expression. **a** Schematic of the strategy to complement the Δ*DPY19* c1 and c2 clones. **b** Southern blot analysis of parental NF54, two clonal Δ*DPY19* parasite lines (c1 and c2) and the complemented Δ*DPY19* Comp clones after digestion of genomic DNA with *EcoR*I/*Cla*I/*Kpn*I. The probe used (orange bar) for genotyping and the expected fragment sizes after digestion of the loci are shown. Data represent n=1 experiment confirming complementation of two independent knockout clones.

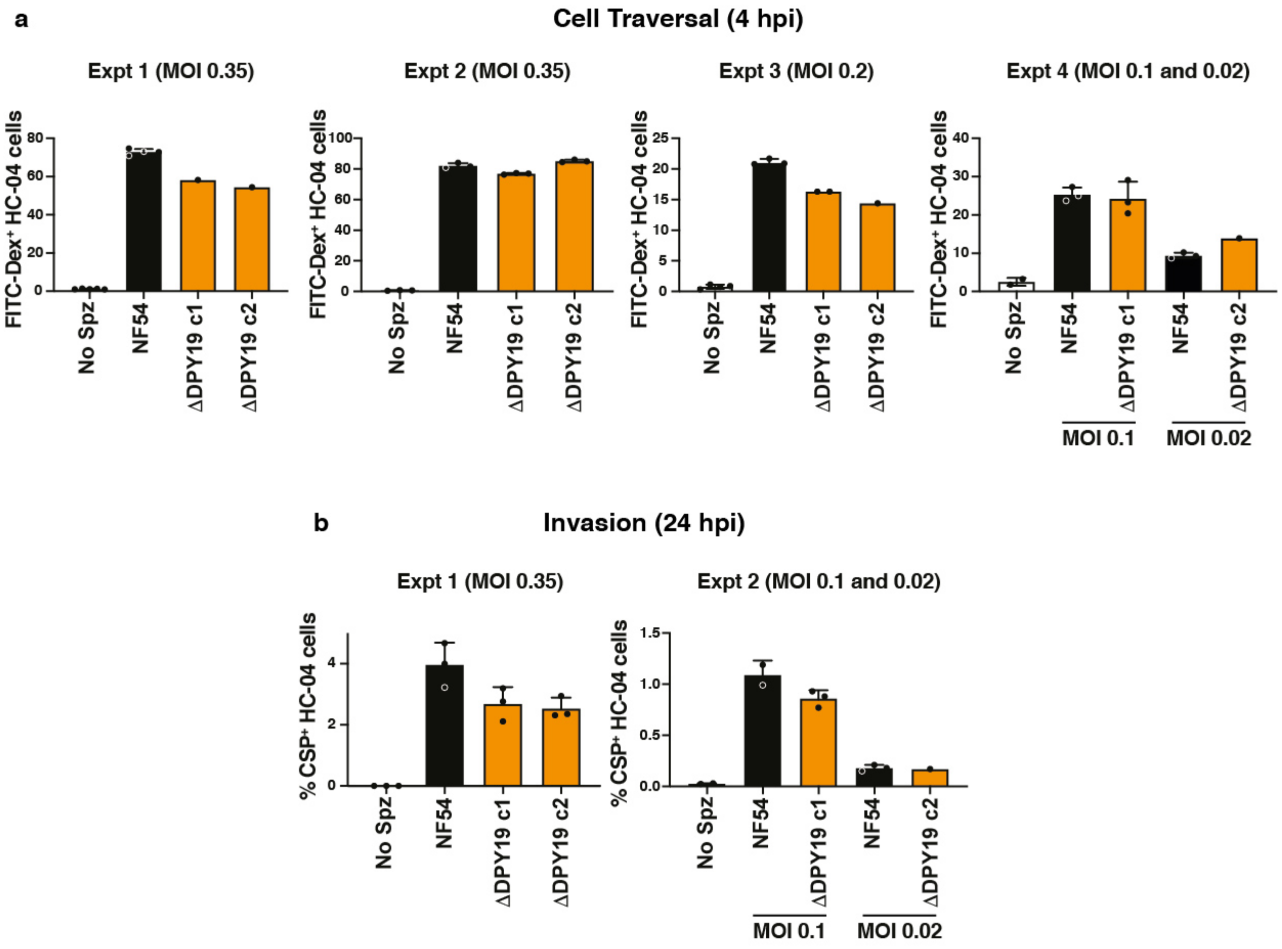

Supplementary Figure 9. Human HC-04 hepatocyte infection assays. **a** Flow cytometric quantification of cell traversal by *P. falciparum* sporozoites after incubation for 4 hours using FITC-dextran uptake to label the wounded cells. **b** Flow cytometric quantification of intracellular *P. falciparum* liver stages after incubation with sporozoites for 24 hours using CSP-positive antibody staining of fixed, permeabilized cells. Variance between assays occurred and was likely due to very low D*DPY19* sporozoite yields from the low oocyst intensities, which restricted the multiplicity of infection to 0.02-0.35, as indicated. The number of biological replicates (wells) in (a) and (b) is shown as data points in each graph. Data are mean ± s.e.m.

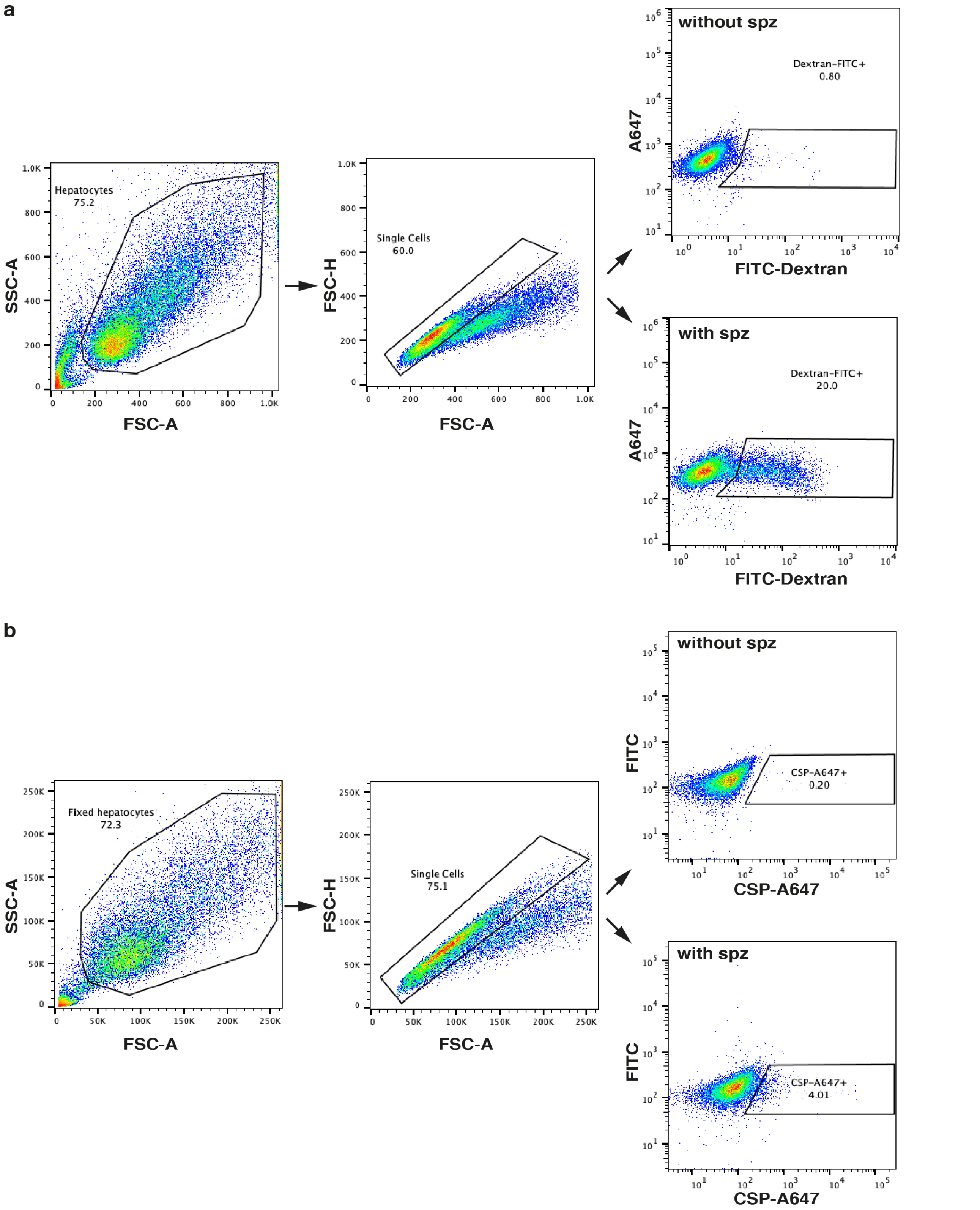

Supplementary Figure 10. Related to Supplementary Figure 9. Flow cytometry gating strategies for HC-04 cell traversal and invasion. **a** Gate 1: HC-04 population identified by forward scatter and side scatter. Gate 2: cells plotted as FSC-H/FSC-A with equivalent height to area ratios to capture single cells only. Gate 3: HC-04 cells plotted against FITC-Dextran/A647 to identify dextran-positive cells. Without sporozoites (spz; control) or with sporozoites (experiment). A647 is invasion control (see b). **b** Gate 1: HC-04 population identified by forward scatter and side scatter. Gate 2: cells plotted as FSC-H/FSC-A with equivalent height to area ratios to capture single cells only. Gate 3: HC-04 cells plotted against CSP-A647/FITC to identify CSP-positive cells. Without sporozoites (spz; control) or with sporozoites (experiment). FITC is traversal control (see a).

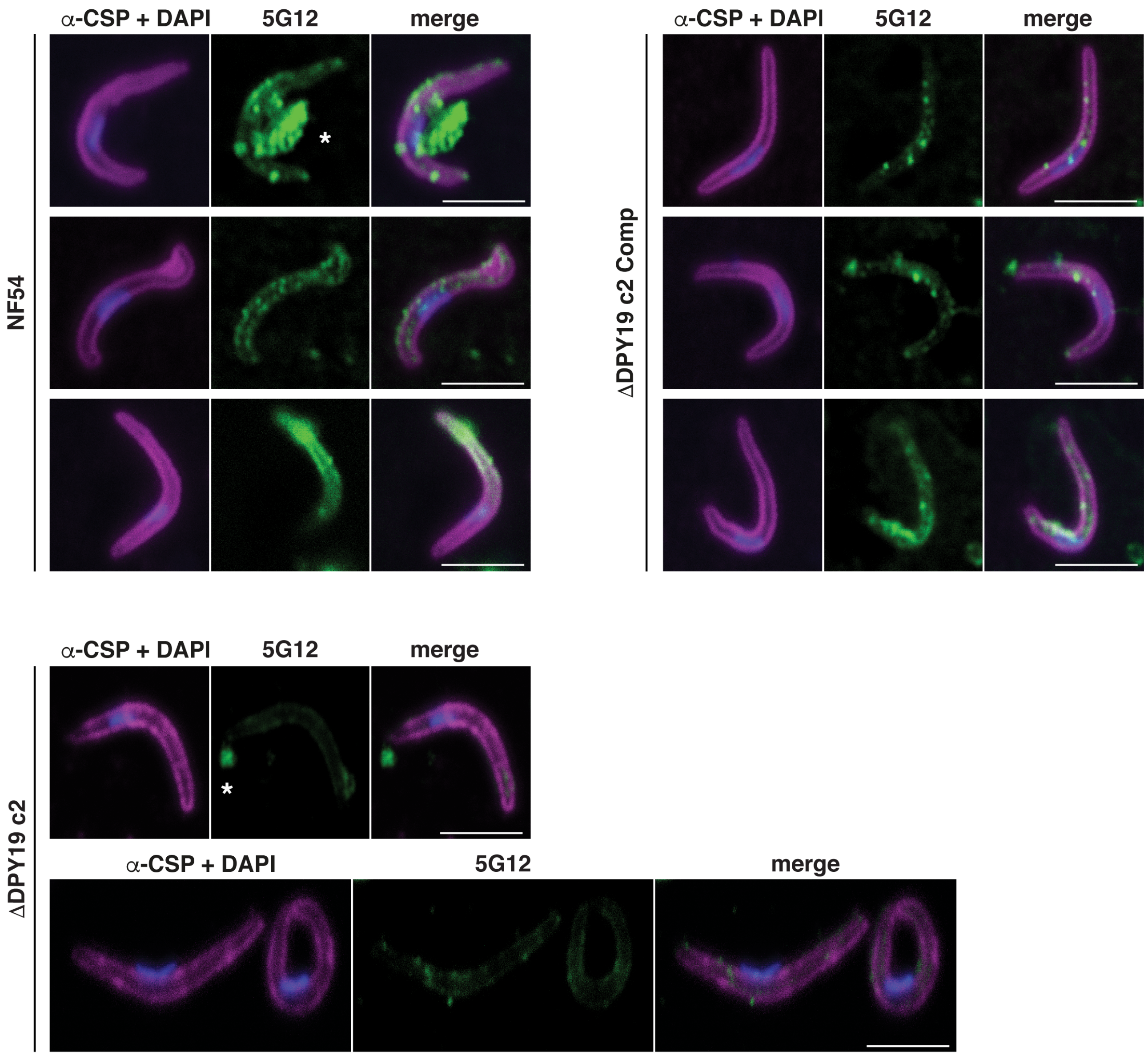

Supplementary Figure 11. Related to Figure 6. Immunofluorescence microscopy of NF54, ΔDPY19 and ΔDPY19 Complemented salivary gland sporozoites with antibodies to CSP (magenta), 5G12 that recognized the EW(Man) epitope (green) and nuclei stained with DAPI. Scale, 5 µm. Cross-reactive material from salivary glands is indicated with an asterisk, noting that uninfected glands also contain C-mannosylated proteins in Figure 6. Data represent n=2 independent experiments.
