# Supplementary material for: Tryptophan C-mannosylation is critical for Plasmodium falciparum transmission
Source: Nat Commun. 2022 Jul 29;13:4400. doi: 10.1038/s41467-022-32076-8 (PMC9338275; doi:10.1038/s41467-022-32076-8)
Supplement: Supplementary file 3 — Description of Additional Supplementary Files [file 41467_2022_32076_MOESM3_ESM.pdf]

Supplementary Data 1: Models for *P. falciparum* proteins with a TSR domain were downloaded from the UniProt database (Q8I2A0, P19597, P16893, C6KT06, C0H4X0, Q8IL45, O97267, O96207, Q8I5M8, Q8IJB7) and the boundaries of predicted globular domains manually assigned. Hitherto unrecognised domains were then classified using structural homology searches with DALI, and those with Z scores >2 were annotated in Fig. 1a and Supplementary Data 1.

Supplementary Movie 1: Full microgameotocyte egress and exflagellation, NF54
Supplementary Movie 2: Partial microgameotocyte egress and exflagellation, NF54
Supplementary Movie 3: Failed egress (no exflagellation), NF54
Supplementary Movie 4: No microgametocyte egress with successful exflagellation, ΔDPY19 c2
Supplementary Movie 5: Failed egress (no exflagellation), ΔDPY19 c2
Supplementary Movie 6: Macrogametocyte egress, NF54
Supplementary Movie 7: Failed macrogametocyte egress, NF54
