# Supplementary material for: Tryptophan C-mannosylation is critical for Plasmodium falciparum transmission
Source: Nat Commun. 2022 Jul 29;13:4400. doi: 10.1038/s41467-022-32076-8 (PMC9338275; doi:10.1038/s41467-022-32076-8)
Supplement: Supplementary file 12 — Reporting Summary [file 41467_2022_32076_MOESM12_ESM.pdf]

nature portfolio

Corresponding author(s): Justin Boddey
Ethan Goddard-Borger

Last updated by author(s): Jun 28, 2022

Reporting Summary

Nature Portfolio wishes to improve the reproducibility of the work that we publish. This form provides structure for consistency and transparency in reporting. For further information on Nature Portfolio policies, see our Editorial Policies and the Editorial Policy Checklist.

Statistics

For all statistical analyses, confirm that the following items are present in the figure legend, table legend, main text, or Methods section.

n/a	Confirmed	
☐	☒	The exact sample size (*n*) for each experimental group/condition, given as a discrete number and unit of measurement
☐	☒	A statement on whether measurements were taken from distinct samples or whether the same sample was measured repeatedly
☐	☒	The statistical test(s) used AND whether they are one- or two-sided<br>*Only common tests should be described solely by name; describe more complex techniques in the Methods section.*
☒	☐	A description of all covariates tested
☒	☐	A description of any assumptions or corrections, such as tests of normality and adjustment for multiple comparisons
☐	☒	A full description of the statistical parameters including central tendency (e.g. means) or other basic estimates (e.g. regression coefficient) AND variation (e.g. standard deviation) or associated estimates of uncertainty (e.g. confidence intervals)
☐	☒	For null hypothesis testing, the test statistic (e.g. *F*, *t*, *r*) with confidence intervals, effect sizes, degrees of freedom and *P* value noted<br>*Give P values as exact values whenever suitable.*
☒	☐	For Bayesian analysis, information on the choice of priors and Markov chain Monte Carlo settings
☒	☐	For hierarchical and complex designs, identification of the appropriate level for tests and full reporting of outcomes
☒	☐	Estimates of effect sizes (e.g. Cohen's *d*, Pearson's *r*), indicating how they were calculated

Our web collection on statistics for biologists contains articles on many of the points above.

Software and code

Policy information about availability of computer code

Data collection	ZEN Black v14.0 (Zeiss)
Data analysis	FIJI software, ImageJ v1.0 (https://imagej.net/Fiji); FlowJo for Mac v10.8, FlowJo LLC; Prism 9 for macOS, GraphPad (https://www.graphpad.com/scientificsoftware/prism/), Adobe Photoshop 2021 v22.4.1, Adobe Illustrator 2021 25.4.

For manuscripts utilizing custom algorithms or software that are central to the research but not yet described in published literature, software must be made available to editors and reviewers. We strongly encourage code deposition in a community repository (e.g. GitHub). See the Nature Portfolio guidelines for submitting code & software for further information.

Data

Policy information about availability of data

All manuscripts must include a data availability statement. This statement should provide the following information, where applicable:

- Accession codes, unique identifiers, or web links for publicly available datasets
- A description of any restrictions on data availability
- For clinical datasets or third party data, please ensure that the statement adheres to our policy

The biological data generated in this study are provided in the Source Data file available in Supplementary Information. Biological tools produced in this study are available from the corresponding authors upon request. Mass spectrometry proteomics data have been deposited to the ProteomeXchange Consortium via the PRIDE partner repository with the dataset identifier PXD033470.

Human research participants

Policy information about studies involving human research participants and Sex and Gender in Research.

Reporting on sex and gender	No human research participants were used in this study.
Population characteristics	*Describe the covariate-relevant population characteristics of the human research participants (e.g. age, genotypic information, past and current diagnosis and treatment categories). If you filled out the behavioural & social sciences study design questions and have nothing to add here, write "See above."*
Recruitment	*Describe how participants were recruited. Outline any potential self-selection bias or other biases that may be present and how these are likely to impact results.*
Ethics oversight	*Identify the organization(s) that approved the study protocol.*

Note that full information on the approval of the study protocol must also be provided in the manuscript.

Field-specific reporting

Please select the one below that is the best fit for your research. If you are not sure, read the appropriate sections before making your selection.

- [x] Life sciences
- [ ] Behavioural & social sciences
- [ ] Ecological, evolutionary & environmental sciences

For a reference copy of the document with all sections, see nature.com/documents/nr-reporting-summary-flat.pdf

Life sciences study design

All studies must disclose on these points even when the disclosure is negative.

Sample size	Sample sizes in this study were chosen based on previously published literature.
Data exclusions	No data were excluded.
Replication	Experiments were repeated. Repeated measures are shown in the Figure and legend.
Randomization	All samples of parasites, mosquitoes, proteins were randomly selected from the available population.
Blinding	Samples for quantification by microscopy were blinded and then unblinded following statistical analyses. Other samples were not blinded as this did not affect the generation or analysis of data.

Reporting for specific materials, systems and methods

We require information from authors about some types of materials, experimental systems and methods used in many studies. Here, indicate whether each material, system or method listed is relevant to your study. If you are not sure if a list item applies to your research, read the appropriate section before selecting a response.

Materials & experimental systems

n/a	Involved in the study	
☐	☒	Antibodies
☐	☒	Eukaryotic cell lines
☒	☐	Palaeontology and archaeology
☐	☒	Animals and other organisms
☒	☐	Clinical data
☒	☐	Dual use research of concern

Methods

n/a	Involved in the study	
☒	☐	ChIP-seq
☐	☒	Flow cytometry
☒	☐	MRI-based neuroimaging

Antibodies

Antibodies used	mouse anti-EW(Man) 5G12 (ref 8) WB, 1:1000; IF, 1:500<br>rabbit anti-PMV R1245 (ref 37) IF, 1:1000<br>rat anti-PTRAMP (ref 39) WB, 1:250<br>mouse anti-HSP70 (ref 67) WB, 1:500

	rabbit anti-MTRAP (ref 70) WB, 1:500<br>rabbit anti-Aldolase (ref 71) WB, 1:4000<br>rabbit anti-TRAP (ref 72) WB, 1:2000<br>mouse anti-CSP 2A10 (ref 73) WB, 1:9000; IF, 1:2000<br>mouse anti-CSP 2A10 conjugated to anti mouse IgG (Alexafluor 647) Invitrogen (in-house) IF, 1:2000<br>mouse anti-Pfs25 4B7 (ref 83) IF, 1:500<br>rabbit anti-SPATR R3855 (in-house) WB, 1:500<br>rat anti-HA 3F10 high-affinity Roche 11867423001 WB, 1:1000; IF, 1:500<br>mouse anti-Tubulin DM1A Sigma T6199 IF, 1:300<br>anti-mouse IgG (HRP) ab97023 WB, 1:1000<br>anti-rabbit IgG (HRP) Merck WB: 1:1000<br>anti-rat IgG (HRP) Merck WB: 1:1000<br>anti-rabbit IgG (Alexa Fluor 594) Invitrogen A110537, Lot# 1420978 IF, 1:1000<br>anti-mouse IgG (Alexa Fluor 594) Invitrogen A11005, Lot# 1310420 IF, 1:1000<br>anti-rat IgG (Alexa Fluor 594) Invitrogen A21471, Lot# 2069713 IF, 1:1000<br>anti-rabbit IgG (Alexa Fluor 488) Invitrogen A1108, Lot# 1829924 IF, 1:1000<br>anti-mouse IgG (Alexa Fluor 488) Invitrogen A11059, Lot# 997757 IF, 1:1000<br>anti-rat IgG (Alexa Fluor 488) Invitrogen A21208, Lot# 1322327 IF, 1:1000<br>anti-HA Affinity Matrix Roche 11815016001 IP, 50 ul, WB, IF (ref 8)
Validation	Antibodies were validated in the references supplied above. SPATR was validated in this study using DOY19 mutants that express less of this protein. HA, HRP, AlexaFluor antibodies were validated in PMID 20130643, Tubulin in PMID 27832590.

Eukaryotic cell lines

Policy information about cell lines and Sex and Gender in Research

Cell line source(s)	HC-04 were not obtained comercially but from the US Naval Medical Research Centre
Authentication	Cells were authenticated by us previously (PMID28355563)
Mycoplasma contamination	Cells were confirmed to be mycoplasma-free
Commonly misidentified lines (See ICLAC register)	No misidentified lines were used in this study.

Animals and other research organisms

Policy information about studies involving animals; ARRIVE guidelines recommended for reporting animal research, and Sex and Gender in Research

Laboratory animals	No laboratory animals were used in this study
Wild animals	No wild animals were used in this study
Reporting on sex	No findings apply to only one sex.
Field-collected samples	No field samples were used in this study
Ethics oversight	Ethics was approved by the Walter and Eliza Hall Institute Biosafety Committee to work with genetically modified malaria parasites and HC-04 cells (NLRD1909).

Note that full information on the approval of the study protocol must also be provided in the manuscript.

Flow Cytometry

Plots

Confirm that:

- [x] The axis labels state the marker and fluorochrome used (e.g. CD4-FITC).
- [x] The axis scales are clearly visible. Include numbers along axes only for bottom left plot of group (a 'group' is an analysis of identical markers).
- [x] All plots are contour plots with outliers or pseudocolor plots.
- [x] A numerical value for number of cells or percentage (with statistics) is provided.

Methodology

Sample preparation	Traversal: Cells were detached with trypsin<br>Invasion: Cells were detached with trypsin, fixed and permeabilized with BD Cytofix/Cytoperm. Monoclonal anti-CSP 2A10

	conjugated to AlexFluor 647 was used for staining and cells were washed twice before analysis.
Instrument	BD Fortessa X20 HTS Reader
Software	FlowJo v10
Cell population abundance	Traversal 10-85%; Invasion 0.2-5%.
Gating strategy	Cells > single cells > fluorescent positive

☒ Tick this box to confirm that a figure exemplifying the gating strategy is provided in the Supplementary Information.
